# Middle East Respiratory Syndrome Coronavirus in Camels, Kazakhstan, 2025

**DOI:** 10.3201/eid3211.260394

**Published:** 2026-11

**Authors:** Aidyn Kydyrmanov, Yermukhammet Kassymbekov, Sardor Nuralibekov, Temirlan Sabyrzhan, Yelizaveta Khan, Marat Kumar, Aigerim Abdimadiyeva, Kobey Karamendin

**Affiliations:** Research and Production Center for Microbiology and Virology, Almaty, Kazakhstan

**Keywords:** Middle East respiratory syndrome coronavirus, MERS, coronavirus, viruses, camels, Central Asia, zoonoses, molecular epidemiology, respiratory infections, whole-genome sequencing, phylogenetic analysis, genomic surveillance, recombination, Kazakhstan

## Abstract

In 2025, a respiratory disease outbreak occurred among camels in Kazakhstan. We detected Middle East respiratory syndrome coronavirus by using quantitative reverse transcription PCR in 38 symptomatic camels, demonstrating active circulation in Central Asia. Our findings highlight the need for enhanced surveillance to assess zoonotic transmission risk.

Middle East respiratory syndrome coronavirus (MERS-CoV) is a zoonotic betacoronavirus primarily found in dromedary camels and capable of causing severe respiratory disease in humans (*1*–*3*). MERS-CoV circulates widely in camel populations in the Arabian Peninsula and Africa (*4*), whereas data from Central Asia remain limited. In Kazakhstan, serologic investigations conducted in 2015 found no evidence of MERS-CoV infection in camelids (*5*). A study published in 2022, detected antibodies reactive to MERS or MERS-like coronaviruses in both Bactrian and dromedary camels, suggesting previous exposure without molecular evidence of active infection (*6*). Molecular testing of 48 asymptomatic camels sampled from the same population in 2020 was negative for MERS-CoV (*7*), and no viral RNA was detected in 13 samples collected from symptomatic camels in the same herd in 2023. In late November 2025, camel herders in the Mangystau region of Kazakhstan reported a respiratory disease outbreak affecting multiple herds. We investigated whether MERS-CoV was circulating during that outbreak.

## The Study

The Mangystau region in southwestern Kazakhstan, which borders Turkmenistan and Uzbekistan, contains approximately one third of the country’s camel population (Figure 1). In western Kazakhstan, camel production primarily involves extensive pastoral and seminomadic systems with seasonal animal movement, mixed dromedary–Bactrian herds, and congregation at shared grazing areas and watering points, which might enable pathogen transmission between herds.

**Figure 1:**
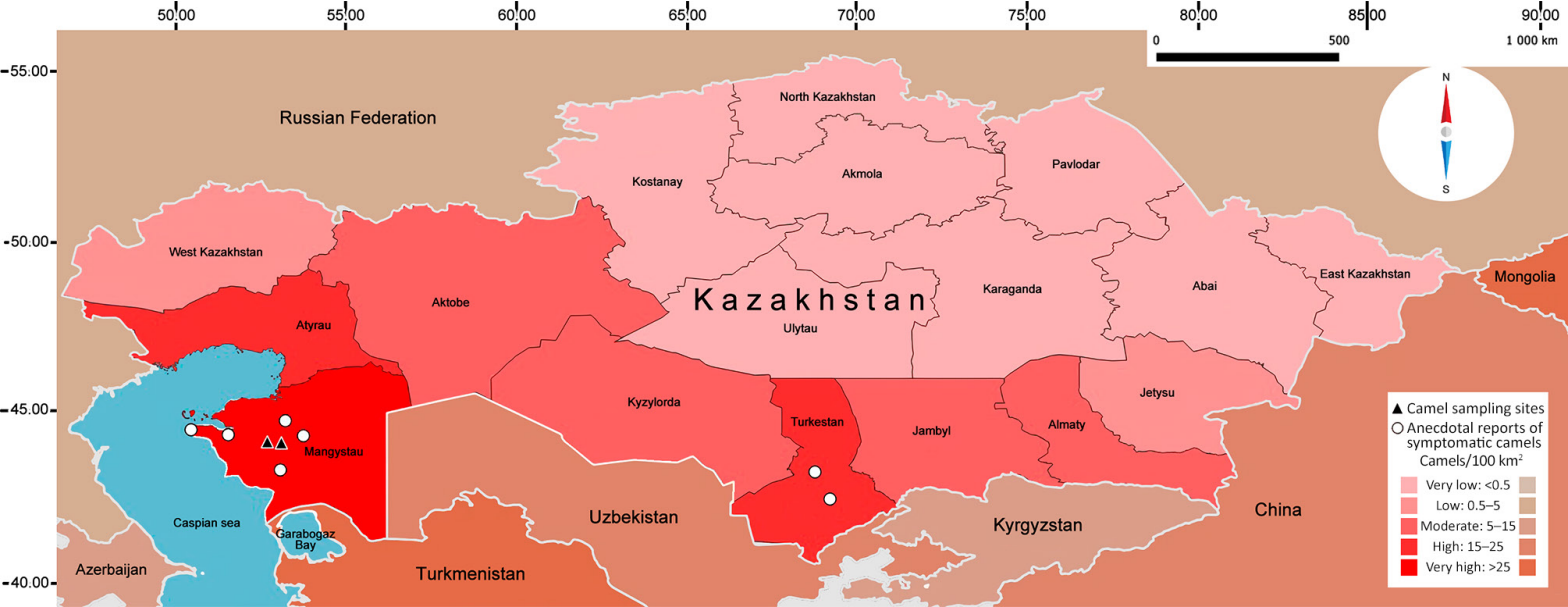
Choropleth representation of camel density used in a study of Middle East respiratory syndrome coronavirus camels, Kazakhstan, 2025. Camel sampling sites and areas with anecdotal reports of symptomatic camels are shown. Colors indicate animals/100 km^2^ in Kazakhstan and surrounding countries. All spatial data processing, density calculations, and map visualization were conducted by using QGIS (version 3.40.12, https://qgis.org). All spatial data were projected in by using the World Geodetic System 1984 (EPSG:4326). Map design displays geographic coordinates so that equal angular intervals of longitude and latitude are represented uniformly, despite corresponding to different ground distances, producing an elongation of the study area.

The outbreak began in late November 2025, when temperatures were below freezing. Affected animals were predominantly dromedaries, although affected Bactrian camels were also found in mixed herds. Clinical signs included lacrimation, nasal discharge, and intermittent cough, which were observed in both juvenile and adult camels. Questionnaire data from camel breeders and veterinary practitioners, indicated similar respiratory disease in multiple areas of Mangystau region, and comparable signs were also reported by camel breeders in Turkistan region.

During December 1–18, 2025, we collected nasopharyngeal swab samples from 38 symptomatic camels from multiple MERS-CoV–positive herds or households in different settlements within the rural district. That sampling accounted for ≈1.2% of the 3,190 camels registered in the affected rural district.

We extracted viral RNA from the nasal swab samples and evaluated for MERS-CoV RNA by using 1-step quantitative reverse transcription PCR (qRT-PCR) targeting the upstream envelope (upE) and open reading frame (ORF) 1a (*8*,*9*). All 38 samples tested positive for MERS-CoV RNA with both assays (Table). Cycle threshold (Ct) values for upE ranged from 23.14–33.09 (median 29.31), and for ORF1a ranged from 14.33–31.09 (median 25.89). Approximately 4 months after the outbreak, we collected follow-up nasopharyngeal swab and serum samples from 14 camels in previously affected herds; all nasopharyngeal swabs were MERS-CoV–negative by qRT-PCR. To evaluate potential causes of infection, we tested samples for influenza A and D viruses, bovine paramyxovirus, and herpesviruses by using molecular assays; all results were negative.

**Table:** Results of quantitative reverse transcription PCR of nasopharyngeal swab samples positive for Middle East respiratory syndrome coronavirus, Kazakhstan, 2025*

Camel species	Age, y	Consistent clinical signs	UpE, Ct	ORF1a, Ct	RdRp, bp	Sequenced†
Dromedary	12	Nasal discharge	27.94	24.13	760	NA
Dromedary	10	Nasal discharge	30.80	26.60	760	NA
Bactrian	<1	Nasal discharge	29.37	25.29	760	NA
Bactrian	6	Nasal discharge	26.90	22.50	760	760-nt RdRp
Dromedary	4	Nasal discharge	34.30	30.13	NA	NA
Dromedary	13	Nasal discharge	33.40	29.78	NA	NA
Dromedary	10	Nasal discharge	31.65	28.25	NA	NA
Dromedary	12	Nasal discharge	27.20	22.30	760	760-nt RdRp and full spike gene
Bactrian	12	Nasal discharge	28.43	25.97	440	440-nt RdRp and full spike gene
Bactrian	8	Nasal discharge	30.60	27.00	440	440-nt RdRp
Bactrian	12	Nasal discharge	28.90	24.70	NA	NA
Dromedary	<1	Nasal discharge	23.56	20.66	760	Whole genome
Dromedary	<1	Nasal discharge	26.55	22.40	760	NA
Bactrian	7	Nasal discharge	31.19	26.41	NA	NA
Dromedary	9	Nasal discharge	28.70	25.10	NA	NA
Dromedary	10	Nasal discharge	29.05	22.22	NA	NA
Bactrian	8	Nasal discharge	32.10	28.02	NA	NA
Dromedary	10	Nasal discharge	23.82	14.33	760	Full spike gene
Dromedary	<1	Nasal discharge	33.09	30.61	760	NA
Dromedary	14	Nasal discharge	28.93	26.63	760	NA
Dromedary	6	Nasal discharge	30.40	27.10	760	NA
Dromedary	<1	Nasal discharge	29.82	26.29	NA	NA
Dromedary	12	Nasal discharge	31.20	27.29	NA	NA
Dromedary	12	Nasal discharge	33.00	29.50	NA	NA
Dromedary	10	Nasal discharge	29.13	25.97	NA	NA
Dromedary	10	Nasal discharge	29.30	25.40	NA	NA
Dromedary	10	Nasal discharge	25.99	22.73	760	NA
Dromedary	14	Nasal discharge	28.60	24.70	760	NA
Dromedary	9	Nasal discharge	23.14	31.09	NA	NA
Dromedary	8	Nasal discharge	32.80	29.32	NA	NA
Dromedary	7	Nasal discharge	30.00	27.47	NA	NA
Dromedary	13	Nasal discharge	33.50	29.59	NA	NA
Dromedary	13	Nasal discharge	31.76	30.46	NA	NA
Dromedary	8	Nasal discharge	28.80	25.10	NA	NA
Dromedary	9	Nasal discharge	26.76	24.41	NA	NA
Dromedary	3	Nasal discharge	25.22	23.25	NA	NA
Dromedary	5	Nasal discharge	29.32	25.33	NA	NA
Dromedary	7	Nasal discharge	28.40	25.72	NA	NA
Median Ct			29.31	25.89	NA	NA

*bp, base pair; Ct, cycle threshold; nt, nucleotide; NA, not applicable; ORF1a, open reading frame 1a; RdRp, RNA-dependent RNA polymerase; UpE, upstream envelope protein. †RdRp was amplified by using pancoronavirus RT-PCR and then selected amplicons were sequenced.

For molecular confirmation, we amplified partial RNA-dependent RNA polymerase gene fragments from a subset of MERS-CoV–positive samples by using pancoronavirus RT-PCR assays (*10*), and sequenced selected amplicons. We performed whole-genome sequencing for 1 representative virus and obtained complete spike gene sequences from 4 outbreak-associated viruses (*11*). Our whole-genome phylogenetic analysis placed the Kazakhstan genome within clade B5, clustering with contemporary camel-derived viruses from Saudi Arabia and Egypt (2023–2024) (Figure 2). Phylogenetic analysis of the 4 complete spike gene sequences placed the viruses from Kazakhstan within the recently described B5-2023.4 sublineage, forming a well-supported cluster with contemporary camel viruses from Saudi Arabia (Figure 3).

**Figure 2:**
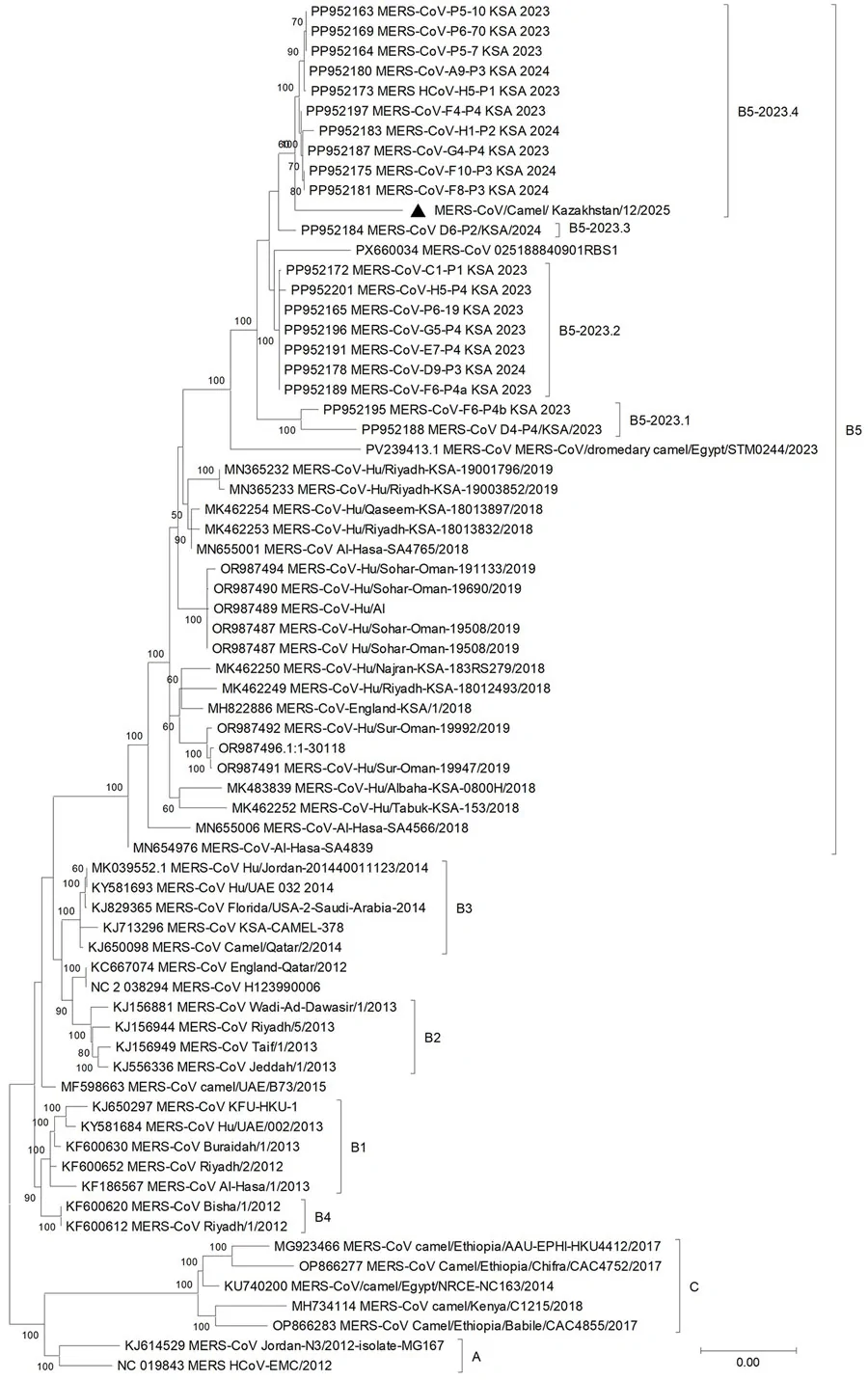
Whole-genome phylogenetic analysis of MERS-CoV detected in camels, Kazakhstan, 2025. Complete genome sequences were aligned by using MAFFT version 7 (https://mafft.cbrc.jp/alignment/software). Maximum-likelihood phylogenetic trees were reconstructed in IQ-TREE (https://iqtree.github.io) by using the best-fit nucleotide substitution model selected by ModelFinder, and branch support was assessed with 1,000 bootstrap replicates. Black triangle indicates MERS-CoV sequence from Kazakhstan (GenBank accession no. PZ439299). Scale bar indicates nucleotide substitutions per site. MERS-CoV, Middle East respiratory syndrome coronavirus.

**Figure 3:**
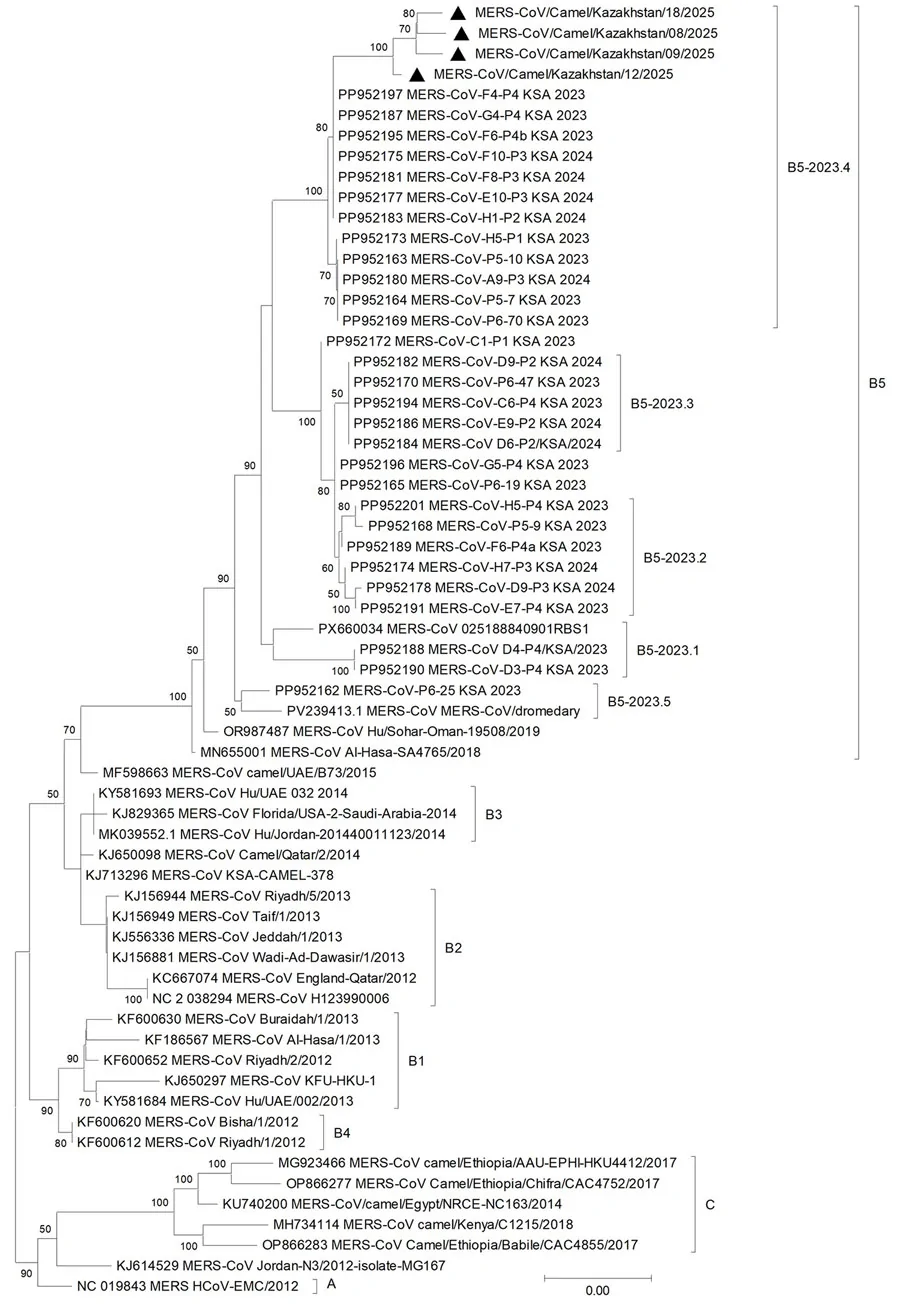
Spike gene phylogenetic analysis of MERS-CoV detected in camels, Kazakhstan, 2025. Maximum-likelihood phylogenetic tree inferred from full-length spike gene nucleotide sequences of representative MERS-CoV strains. Sequences were aligned by using MAFFT version 7 (https://mafft.cbrc.jp/alignment/software) and the phylogeny was reconstructed by using IQ-TREE (https://iqtree.github.io). Black triangles indicated sequences from Kazakhstan obtained in this study (GenBank accession nos. PZ439299, PZ450434, PZ450435, PZ450436). The Kazakhstan sequences clustered within clade B5 along with contemporary camel-derived strains from the Arabian Peninsula. Numbers at nodes indicate percent bootstrap support values on the basis of 1,000 replicates. Scale bar indicates nucleotide substitutions per site. MERS-CoV, Middle East respiratory syndrome coronavirus.

Comparison with recently available complete MERS-CoV genomes showed that the viruses from Kazakhstan shared spike protein substitutions P97L, G198D, and L495P with contemporary B5-2023.4 viruses, although we did not identify the L450F substitution among the publicly available genomes analyzed. Both L450F and L495P substitutions were located within the receptor-binding domain.

We performed a recombination analysis by using Recombination Detection Program version 4.101 (http://web.cbio.uct.ac.za/∼darren/rdp.html), which identified 2 putative recombination events involving the Kazakhstan genome that were supported by multiple independent detection methods (Appendix 1). The inferred recombinant regions overlapped those previously reported among contemporary B5 viruses from Saudi Arabia, and 1 inferred parental lineage could not be assigned with certainty (Appendix 2). Our findings are consistent with the recurrent within-lineage recombination previously described (*12*). However, additional complete genome sequences are required to determine the frequency and evolutionary significance of those recombination events in Central Asia.

## Conclusions

We provided molecular evidence of active MERS-CoV infection in camels in Kazakhstan, extending the documented geographic range of virus circulation into Central Asia. Earlier regional investigations detected serologic reactivity without RNA confirmation (*6*), and nationwide screening conducted during 2020–2021 did not find MERS-CoV antibodies in sampled camels (*7*). The detection of MERS-CoV RNA during the 2025 outbreak suggests active infection in camel populations in Kazakhstan. That detection also likely reflects targeted sampling of acutely symptomatic animals exhibiting extensive nasal discharge and coughing, with clinical signs occasionally linked to MERS-CoV, particularly in younger camels (*9*).

We detected MERS-CoV RNA in both juvenile and adult camels. Although viral RNA is often detected more frequently in juveniles in endemic settings, adult camels remain susceptible to infection and reinfection, with differences in detection patterns likely reflecting levels of preexisting immunity and duration of viral shedding (*13*). The occurrence of infection across multiple age groups in this outbreak is consistent with active virus transmission within affected herds. Together, those findings indicate that active MERS-CoV infection occurs in camel populations in Kazakhstan and highlight the need for expanded surveillance to better define its epidemiology.

Our whole-genome and spike gene phylogenetic analyses placed the viruses from Kazakhstan within the contemporary B5-2023.4 sublineage of MERS-CoV circulating among camels in the Arabian Peninsula. Previous studies have suggested enhanced replication and transmission fitness for some clade B MERS-CoV lineages from the Arabian Peninsula (*14*). Although the viruses from Kazakhstan clustered with contemporary Arabian B5 lineage viruses, the available genomic data do not permit inference of viral phenotype or transmissibility. Detection of a closely related lineage in a region with limited molecular evidence of active MERS-CoV circulation highlights the need for continued surveillance and genomic characterization.

Our genomic analyses revealed that most spike protein substitutions identified in the viruses from Kazakhstan have previously been reported among contemporary B5-2023.4 viruses, whereas the L450F substitution was not identified among the publicly available genomes included in our analysis. Recombination analysis suggested 2 putative recombination events. However, the biologic and evolutionary implication of those findings require further investigation.

Although the available sequence data do not permit determination of the geographic origin or directionality of virus movement, the Mangystau region lies within regional livestock movement networks connecting Central Asia with neighboring regions. Animal movement, livestock exchange, and increasing connectivity with the Arabian Peninsula might enable transboundary dissemination of zoonotic pathogens. Those findings highlight the need for coordinated surveillance and genomic monitoring of camel populations and exposed persons across Central Asia.

The outbreak occurred during winter conditions, in contrast to seasonal peaks often reported in warmer months in endemic regions (*15*). Whether climatic conditions influence viral stability, host susceptibility, or transmission dynamics in temperate continental environments remain to be investigated.

In conclusion, detection of active viral shedding in camels within a high-density livestock region bordering several countries emphasizes the potential for zoonotic spillover. No human MERS cases were identified in connection with the 2025 camelid outbreak in Kazakhstan. However, continued molecular surveillance and expanded genomic characterization of camel MERS-CoV are needed to better define viral diversity, transmission dynamics, and potential public health risks in the region. 

Appendix 1 Additional information about Middle East respiratory syndrome coronavirus camels, Kazakhstan, 2025.

Appendix 2 Inferred recombinant regions of Middle East respiratory syndrome coronavirus camels, Kazakhstan, 2025.
