# Supplementary material for: Middle East Respiratory Syndrome Coronavirus in Camels, Kazakhstan, 2025
Source: Emerg Infect Dis. 2026 Nov;32(11):1907–12. doi: 10.3201/eid3211.260394 (PMC13715869; doi:10.3201/eid3211.260394)
Supplement: Supplementary file 1 [file 26-0394-Techapp-s1.pdf]

Article DOI: https://doi.org/10.3201/eid3211.260394

EID cannot ensure accessibility for supplementary materials supplied by authors. Readers who have difficulty accessing supplementary content should contact the authors for assistance.

Middle East Respiratory Syndrome Coronavirus in Camels, Kazakhstan, 2025

Appendix 1

Additional Methods

Recombination screening was performed with the RDP, GENECONV, BootScan, MaxChi, Chimaera, SiScan and 3Seq methods implemented in RDP v4.101, using the automated exploratory analysis mode. Putative events were accepted only if they were supported by at least three different detection methods with Bonferroni-corrected P values <0.05 and if the inferred breakpoint region and parental assignments were consistent with separate maximum-likelihood phylogenies of the putative recombinant, minor parent and major parent segments. Events flagged by RDP4 as potentially attributable to other evolutionary processes ("~") or with undetermined breakpoints ("*") were treated as tentative and are reported but interpreted with caution.

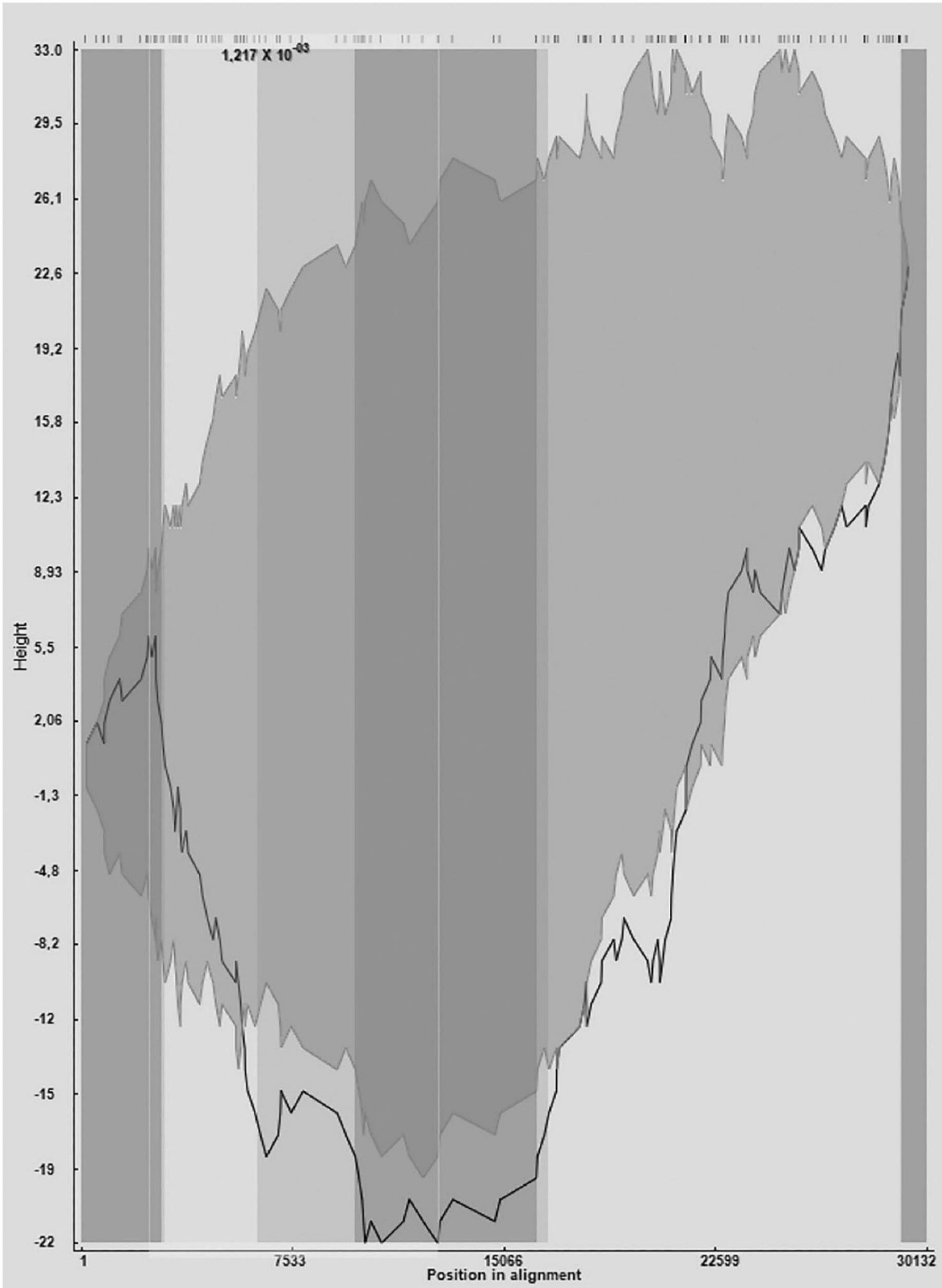

Appendix Figure 1. The plot shows the distribution of Bonferroni-corrected P values across the genome alignment for the different detection methods implemented in RDP v4.101, with the x-axis indicating nucleotide position in the alignment and the y-axis showing test statistic values. Shaded vertical bands denote the inferred breakpoint region (positions 2432–12,726 in the alignment), with the putative recombinant MERS-CoV/Camel/_Kazakhstan/12/2025 and its inferred major (PP952183_MERS-CoV-H1-P2_KSA_2024) and minor ("unknown," proxied by PP952188_MERS-CoV_D4-P4/KSA/2023) parental sequences highlighted in the accompanying schematic. Events marked with the '~' symbol in the RDP4 output (Appendix 2 Table) indicate that the signal could potentially arise from evolutionary processes other than recombination and are therefore considered provisional.

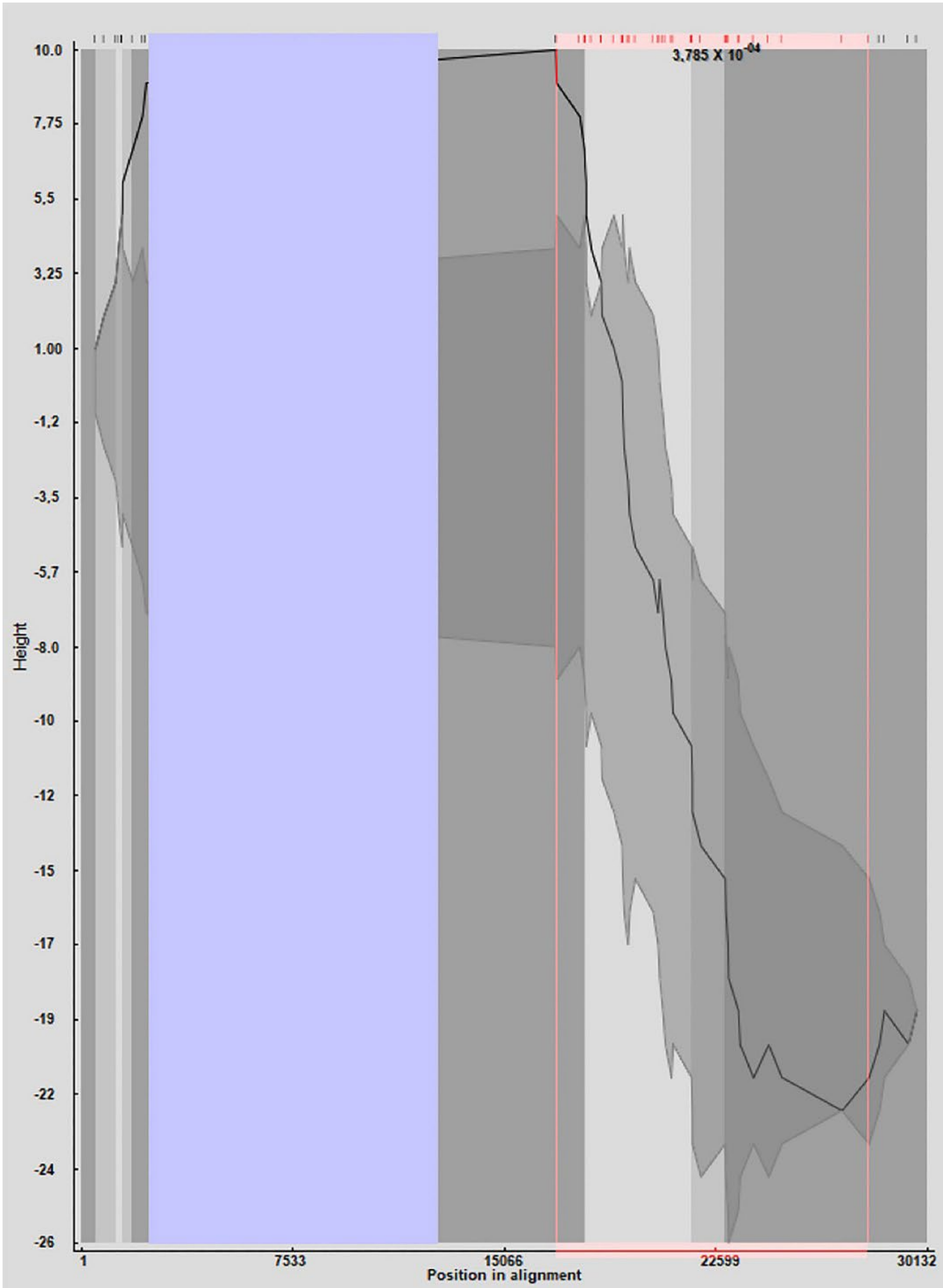

Appendix Figure 2. RDP4 recombination signal and breakpoint mapping for recombination event 5 in MERS-CoV/Camel/_Kazakhstan/12/2025. The plot summarizes support from the different RDP4 methods across the alignment, with breakpoint positions inferred around 16,904–27,989. The recombinant sequence MERS-CoV/Camel/_Kazakhstan/12/2025 is shown together with its inferred minor parent PP952169_MERS-CoV-P6–70_KSA_2023 and major parent PP952201_MERS-CoV-H5-P4_KSA_2023, illustrating that the mid-to-3′ genomic region of the Kazakhstan strain is a mosaic derived from multiple B5-lineage camel viruses. As for event 4, only events supported by ≥3 methods with Bonferroni-corrected $p < 0.05$ and consistent phylogenetic clustering were considered robust, and signals flagged by RDP4 as potentially non-recombinant were interpreted cautiously.

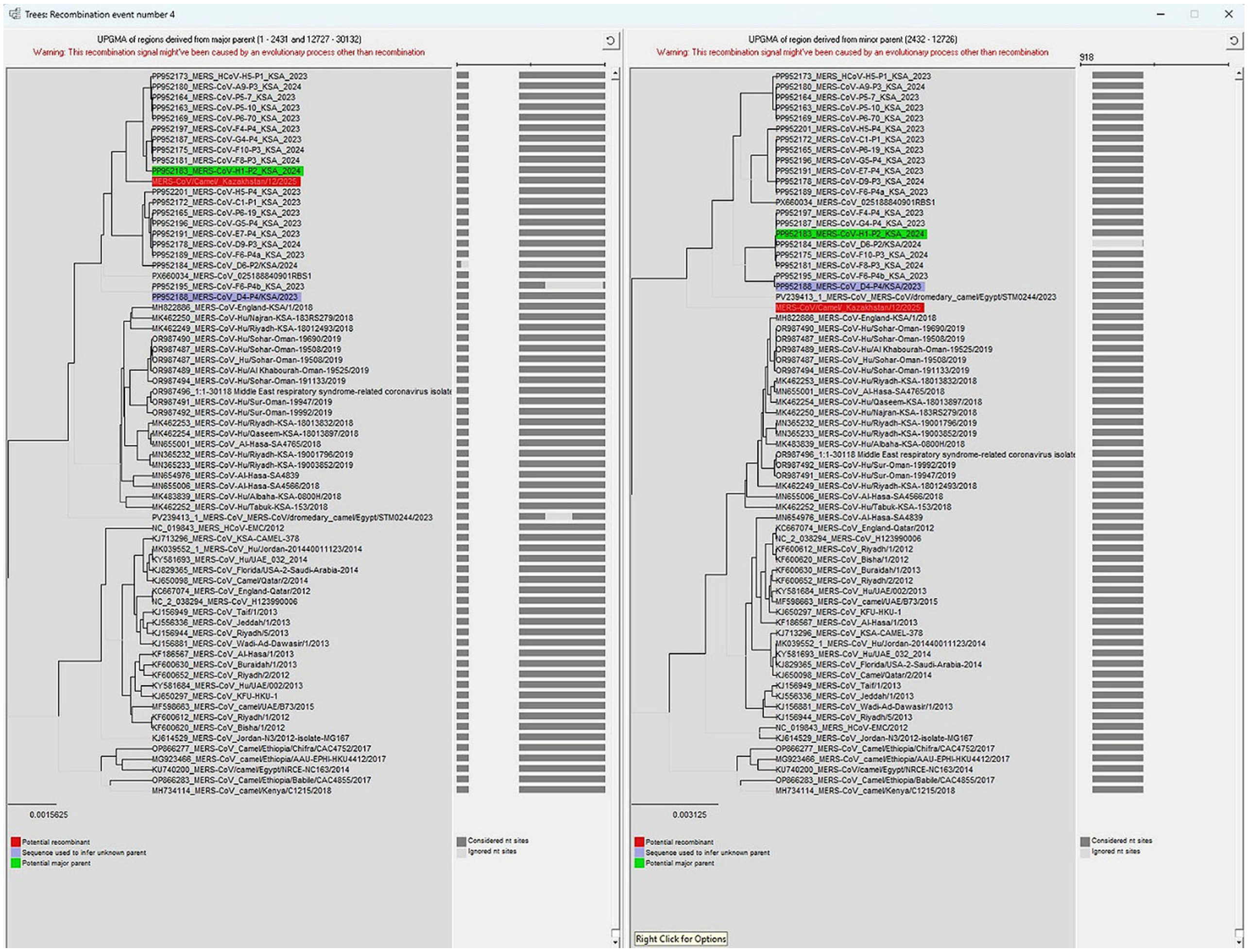

Appendix Figure 3. UPGMA trees inferred from the genomic regions corresponding to the putative major-parent (positions 1–2431 and 12,727–30,132) and minor-parent (positions 2432–12,726) segments are shown side-by-side, with MERS-CoV/Camel/_Kazakhstan/12/2025 (red) clustering with PP952183_MERS-CoV-H1-P2_KSA_2024 (green, major parent) in the major-parent trees and with PP952188_MERS-CoV_D4-P4/KSA/2023 (purple, proxy for an unknown minor parent) in the minor-parent trees. Gray bars to the right indicate alignment sites considered in each segment, and RDP4 warnings about possible alternative evolutionary explanations are retained to emphasize that this event is interpreted as a provisional recombination signal.

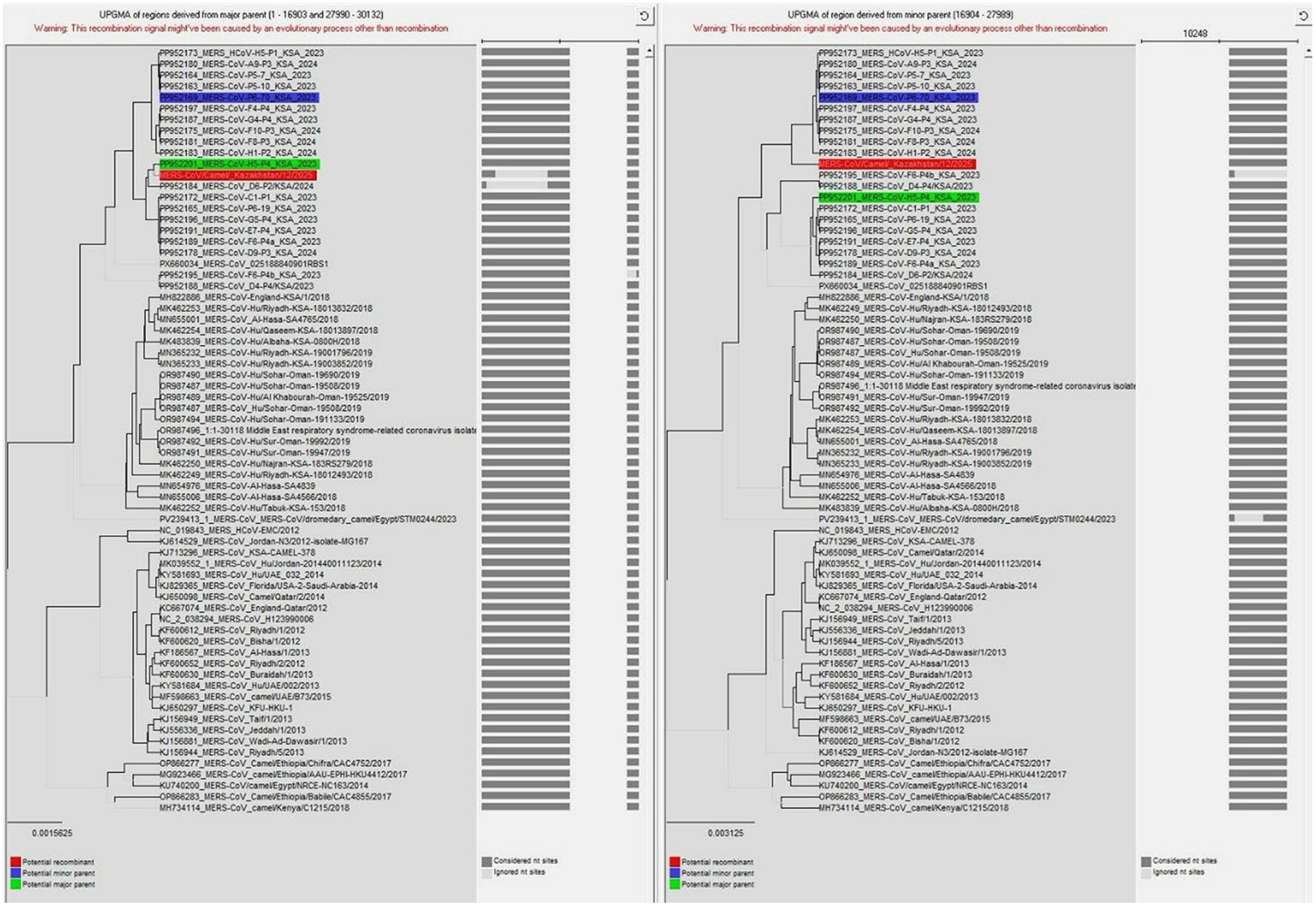

Appendix Figure 4. UPGMA trees derived from genomic regions inherited from the major parent (PP952201_MERS-CoV-H5-P4_KSA_2023, green) and minor parent (PP952169_MERS-CoV-P6–70_KSA_2023, purple) show that MERS-CoV/Camel/_Kazakhstan/12/2025 (red) switches phylogenetic placement between the two segments, consistent with the mosaic structure inferred by RDP v4.101. Vertical gray bars represent the positions included in each segment, and asterisks in the underlying RDP4 table indicate that the true breakpoints may be imprecise because of overlapping recombination events.
